# A cross-sectional ecological study of spatial scale and geographic inequality in access to drinking-water and sanitation

**DOI:** 10.1186/s12939-014-0113-3

**Published:** 2014-11-26

**Authors:** Weiyu Yu, Robert ES Bain, Shawky Mansour, Jim A Wright

**Affiliations:** Geography and Environment, University of Southampton, University Road, Southampton, SO17 1BJ UK; UNICEF, 3 UN Plaza, New York, USA; Geography and GIS Department, Faculty of Arts, Alexandria University, Alexandria, Egypt

**Keywords:** Drinking water, Sanitation, Census, Geographic information systems

## Abstract

**Introduction:**

Measuring inequality in access to safe drinking-water and sanitation is proposed as a component of international monitoring following the expiry of the Millennium Development Goals. This study aims to evaluate the utility of census data in measuring geographic inequality in access to drinking-water and sanitation.

**Methods:**

Spatially referenced census data were acquired for Colombia, South Africa, Egypt, and Uganda, whilst non-spatially referenced census data were acquired for Kenya. Four variants of the dissimilarity index were used to estimate geographic inequality in access to both services using large and small area units in each country through a cross-sectional, ecological study.

**Results:**

Inequality was greatest for piped water in South Africa in 2001 (based on 53 areas (N) with a median population (*MP*) of 657,015; *D* = 0.5599) and lowest for access to an improved water source in Uganda in2008 (N = 56; *MP* = 419,399; *D* = 0.2801). For sanitation, inequality was greatest for those lacking any facility in Kenya in 2009 (N = 158; *MP* = 216,992; *D* = 0.6981), and lowest for access to an improved facility in Uganda in 2002 (N = 56; *MP* = 341,954; *D* = 0.3403). Although dissimilarity index values were greater for smaller areal units, when study countries were ranked in terms of inequality, these ranks remained unaffected by the choice of large or small areal units. International comparability was limited due to definitional and temporal differences between censuses.

**Conclusions:**

This five-country study suggests that patterns of inequality for broad regional units do often reflect inequality in service access at a more local scale. This implies household surveys designed to estimate province-level service coverage can provide valuable insights into geographic inequality at lower levels. In comparison with household surveys, censuses facilitate inequality assessment at different spatial scales, but pose challenges in harmonising water and sanitation typologies across countries.

## Introduction

Following the expiry of the Millennium Development Goals (MDGs) in 2015, inequality in access to services is increasingly considered an important dimension to international monitoring arrangements post-2015 [1,2], especially in light of the recognition of the Human Right to Water and Sanitation [3,4]. A series of studies have shown that one of the most pronounced inequalities in access to improved drinking-water and sanitation relates to location, typically measured in terms of urban versus rural areas. This is apparent, for example, in an analysis of 19 Latin American and Caribbean countries, which showed that location was the circumstance that resulted in the greatest gap in access to improved water for 17 out of 19 countries [5]. Similarly, for sanitation, location resulted in the greatest gap in access for 15 out of 19 countries. In addition, concern has been expressed as to how best to distinguish rural from urban and the consequences of this distinction for international monitoring. It has been suggested for example that the current rural-urban classifications in many countries place rapidly growing peri-urban areas in the ‘rural’ category, thereby leading to an over-estimate of urban coverage in water and sanitation provision and a corresponding under-estimate in rural areas [6].

International monitoring of water and sanitation access is primarily based on a combination of household survey (such as Demographic and Health Surveys and Multiple Indicator Cluster Surveys) and census data [7]. Recent analyses of related inequalities have drawn on household survey rather than census data [8,9]. Although interest in inequality in water and sanitation has only recently received attention as a result of the debate surrounding post-MDG international monitoring arrangements and the Human Right to Water and Sanitation [10], there is a much longer history in the measurement of spatial inequality in health outcomes and deprivation. Evidence from these other domains suggests generally that greater inequality is apparent in more geographically disaggregated data [11–13].

Given the relatively long history of measuring spatial inequality in deprivation and health outcomes, this paper draws on a metric widely used in both of these areas in measuring ethnic segregation, the dissimilarity index. Several variants of this index are applied through an ecological study to census data concerning water and sanitation on population groups within five countries, so as to better understand patterns of inequality in service access. In so doing, the study also aims to understand the issues and potential limitations surrounding the application of the dissimilarity index and its variants to water and sanitation access. It also aims to assess the potential role of census data in understanding geographic inequalities relating to water and sanitation.

## Methods

### Study countries and data

Study countries were selected that were classified as either low or middle income by the World Bank, and for which geographically disaggregated census statistics on water and sanitation were readily available. We selected exemplar countries for which statistics were available for areas with a median population size of 200,000 or lower. Tables 1 and 2 summarise the characteristics of the data used for each country.

**Table 1:** **Characteristics of data describing spatial variation in water and sanitation access in five countries**

**Country**	**Colombia**	**Egypt**	**Kenya**	**South Africa**	**Uganda**
**Data source**	Departmento Administrativo Nacional de Estadistica (DANE) [14]; Humanitarian Response [15]	The Egyptian Cabinet Information and Decision Support Center (IDSC) [16]	Kenya Open Data [17,18]	Statistics South Africa (Stats SA) [19,20]	World Resources Institute (WRI) [21]; Global Administrative Areas (GADM) [22]
**Temporal Coverage**	2005	2006	2009	2001	2002^b^; 2008^a^
**Most disaggregated geography (total number of areal units)**	urban and rural areas within municipalities (2,195)	urban (*Kism*) and rural areas (*Markaz*) within governorates (335 ^a^; 341 ^b^)	sub-location (7,129)	sub-place (20,784)	sub-county (852)
**Median population per smallest areal unit (range)**	5,914 (64 – 6,824,510)	177,798 (21 – 1,169,192)^a^; 175,665 (21 – 1,169,192)^b^	3,734 (7 – 140,321)	795 (1 – 131,659)	28,059 (3,094 – 172,564)^a^; 23,458 (2,430 – 136,322)^b^
**Median population density, people/km** ^**2**^ **(range)**	190.34 (0.10 – 168,723.80)	1,859.80 (0.03 – 80,011.59)^a^; 1,841.88 (0.03 – 80,011.59)^b^	--	431.31 (0.01 – 106,108.80)	190.00 (4.14 – 3,249.47)^a^; 157.70 (3.51 – 2,567.00)^b^
**Percentage of lowest level areal units with missing data**	0.01%	0.07%^a^; 0.05%^b^	0.00%	0.02%	0.12% (rural only)
**Geographic information**	Boundaries data; urban/rural codes	Boundaries data; urban/rural codes	No spatial information available	Boundaries data; urban/rural codes	Boundaries data; rural areas only

Area counts exclude water bodies and those with missing data; ^a^is for water data; and ^b^is for sanitation data.

**Table 2:** **‘Preferred’ and ‘restricted’ water and sanitation access definitions for five case study countries**

**Country**	**Colombia**	**Egypt**	**Kenya**	**South Africa**	**Uganda** ^*^
**‘Preferred’ water access categories**	Piped water in house/on premises; public standpipe; wells with/without pump; rainwater	Access to public drinking water network (tapped water)	Piped; jabia/rainwater tank; borehole; well; spring	Piped water for domestic use	Piped (tap) water; rainwater; gravity flow schemes; boreholes; protected well/spring
**‘Restricted’ water access categories**	Bottled water, tanker, surface waters	No access to public drinking water network (pump; well; other unknown water accesses)	Pond; dam; lake; stream; water vendor; other	No access to piped water for domestic use	Open water source (pond; stream; lake; water hole; unprotected spring; swamp; shallow well); water truck/vendor; other
**‘Preferred’ sanitation access categories**	Access to own sanitation facility in house	Accessible local/public sanitation; trench sanitation system	Piped sewer system; septic tank; pit latrine; ventilated improved pit (VIP) latrine; cesspool	Hygienic toilets (flush toilet; chemical toilet; VIP latrine)	Pit latrine; VIP latrine; flush toilet
**‘Restricted’ sanitation access categories**	Shared sanitation; no access to sanitation	No access to any sanitation system	Bucket; bush; other	Unhygienic toilets (Pit latrine without ventilation; bucket latrine; other; none)	Uncovered pit latrine; bush; other; none

^*^Based on an inventory of water and sanitation services [23].

With the exception of Uganda, all statistics were derived from population censuses. We used census data in preference to household survey data, since given their complete (or near complete) enumeration of population, census data allow much greater geographic disaggregation, whereas household surveys are often only powered to provide statistically robust estimates of improved drinking-water and sanitation coverage at province level. For rural Uganda, water and sanitation coverage statistics were derived by the Directorate of Water Development from an inventory of water and sanitation facilities. Each facility was assumed to provide for a certain number of households, so for example, a deep borehole with handpump was assumed to serve 300 people [23]. The Ugandan population served by such facilities was calculated based on this assumption and then expressed as a proportion of the projected population from the 2002 census. We include this data set so as to explore the utility of censuses of water and sanitation services, alongside population censuses.

### Analysis

On the basis of the census data (or water source inventory data for Uganda), we then classified sources following the JMP classification as far as possible [24]. Given that water and sanitation classes in available census data varied by country and did not always match this JMP classification, we combined the census classes together, so as to approximately distinguish between different JMP classes (see Table 2). We thus distinguish between a higher level of ‘preferred’ water and sanitation and a lower level of ‘restricted’ water and sanitation, but on a country-by-country basis. Percentage coverage figures for households were converted to percentage coverage figures for population by assuming household size was constant within each areal unit.

As a measure of inequality, we chose to use the dissimilarity index, since this is commonly used as a health inequality metric [25,26]. The index is also currently incorporated into the World Bank’s Human Opportunity Index (HOI), a composite measure of progress in service provision based on both coverage and inequality in access [5]. In its simplest form, the dissimilarity index *D* measures the spatial pattern in two population sub-groups, in this case those with and without the drinking-water or sanitation services shown in Table 2. We used the classic formulation of the index, *D* [27]. This measures the proportion of people in the overall population who would have to change location for drinking-water or sanitation access to be completely evenly distributed throughout all areas, based on the following formula:

1 $$ D=\frac{1}{2}{\displaystyle \sum_i}\left|\frac{b_i}{B}-\frac{w_i}{W}\right| $$

where *b*_*i*_ and *B* represent the population without access to either water or sanitation in areal unit *i* and the study area as a whole respectively, whilst *w*_*i*_ and *W* represent the population with access to these services in areal unit *i* and the entire study area. Thus, a *D* value of zero indicates no inequality, whilst a value of 1 indicates complete inequality. Where census data are used, it is generally considered inappropriate to undertake significance testing for *D* since the data represent a complete enumeration of population [28].

The classic dissimilarity index takes no account of the spatial configuration of areal units, comparing service provision to nationwide coverage. However, public awareness of inequalities can be greater where there are highly localised differences in service provision and living standards [29]. We therefore further calculated a neighbourhood-adjusted variant of *D* [30], which assesses more localised variation in service access:

2 $$ D(adj)=D-\frac{{\displaystyle {\sum}_i}{\displaystyle {\sum}_j}\left|{C}_{ij}\left({Z}_i-{Z}_j\right)\right|}{{\displaystyle {\sum}_i}{\displaystyle {\sum}_j}{C}_{ij}} $$

where *z*_*i*_ and *z*_*j*_ are the proportions of population with access to services in areal units *i* and *j* respectively and *c*_*ij*_ is a binary spatial weight which is one when areal units i and j are neighbours and zero otherwise. *D(adj)* thus takes a lower value when access to services is regionally concentrated.

Since some of the most disaggregated areal units in our acquired data are very small (e.g. 2,366 subplaces in South Africa have populations of less than 200), we also calculated two further variants of the classic index that account for random variation in proportions when areal units have small populations, namely Winship’s [31] *D*_*a*_ index and Voas and Williamson’s [32] *D*_a_*. For each of our selected countries, we calculated each of these dissimilarity indices for areal units of differing sizes (e.g. province, district, and sub-district), so as to measure inequalities in service access at different geographic scales. For Kenya, only the non-spatial forms of the dissimilarity index were calculated, since no corresponding boundary data were available for 2009 census files.

To enable a closer examination of inequality in service access between rural, urban and peri-urban areas, we then calculated the local version of dissimilarity index, disaggregated from the traditional *D* index:

3 $$ {d}_i=\frac{1}{2}\left|\frac{b_i}{B}-\frac{w_i}{W}\right| $$

Given *n* as the total count of areal unit within the entire study area, we then defined a threshold value for identifying the contribution to national level inequality:

4 $$ d(t)=\frac{D}{n}=\frac{1}{2n}{\displaystyle \sum_i}\left|\frac{b_i}{B}-\frac{w_i}{W}\right| $$

Since *D*/*n* represents the average areal contribution towards national inequality, an areal unit with value *d*_*i*_ higher than *d(t)* can be considered as a strong contributor towards national inequality of the entire study area, whilst *d*_*i*_ value less than *d(t)* represents a weaker contribution towards national inequality.

To better understand the spatial variations in the inequality measure at different scales, the local *d*_*i*_ index can be decomposed to different geographical levels following the logic of Wong [33]. For instance, assume there are only two geographical levels for the study area, a regional level and a more disaggregated local level. The *d*_*i*_ index of local unit *i* within region *j* can be formulated as:

5 $$ d{(l)}_{ij}=\frac{1}{2}\left|\frac{b_{ij}}{B}-\frac{w_{ij}}{W}\right| $$

where *b*_*ij*_ and *w*_*ij*_ represent the populations with and without access to service respectively in local unit *i* within region *j* respectively. If region *j* is denoted as *R*_*j*_, the local *d*_*i*_ of region *j* is:

6 $$ d{(r)}_j=\frac{1}{2}\left|\frac{{\displaystyle {\sum}_{i\in {R}_j}}{b}_{ij}}{B}-\frac{{\displaystyle {\sum}_{i\in {R}_j}}{w}_{ij}}{W}\right| $$

Accordingly, the threshold *d(t)* can be reframed locally and regionally as the following equations (7) and (8), in order to determine the localised contribution to national level inequality for different scales separately:

7 $$ d{(t)}_l=\frac{1}{2{n}_i}{\displaystyle \sum_j}{\displaystyle \sum_{i\in {R}_j}}\left|\frac{b_{ij}}{B}-\frac{w_{ij}}{W}\right| $$

and

8 $$ d{(t)}_r=\frac{1}{2{n}_j}{\displaystyle \sum_j}\left|\frac{{\displaystyle {\sum}_{i\in {R}_j}}{b}_{ij}}{B}-\frac{{\displaystyle {\sum}_{i\in {R}_j}}{w}_{ij}}{W}\right| $$

where *n*_*i*_ and *n*_*j*_ represent the total number of local units and regions respectively within the entire study area.

The contribution to national level inequality arising purely at the local scale in the *j*th region, *C(l)*_*j*_, excluding any regional contribution, can therefore be formulated as:

9 $$ C{(l)}_j={\displaystyle \sum_{i\in {R}_j}}d{(l)}_{ij}-d{(r)}_j $$

The value of *C(l)*_*j*_ can be positive, negative, or zero. A *C(l)*_*j*_ value of zero indicates overall no inequality contributed purely at the local scale within region *j*. An areal unit with a *C(l)*_*j*_ value greater than zero indicates a strong contributor to inequality locally, whilst a value less than zero indicates a weaker contribution. These localised index values, including *C(l)*_*j*_ can be mapped.

## Results

### Access to drinking-water and sanitation

**Figure 1:**
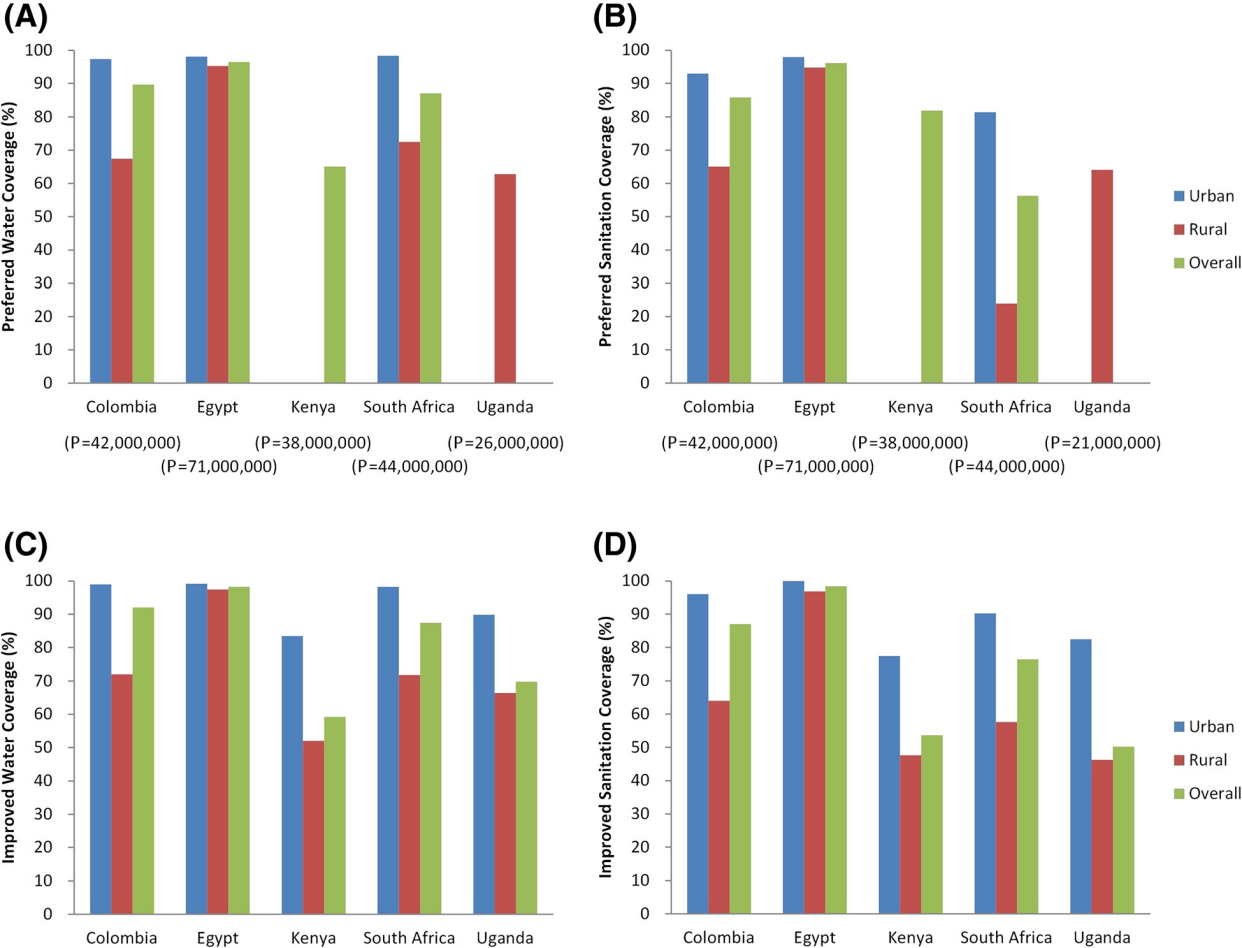
**Percentage water and sanitation coverage by country, broken down by rural versus urban (A, B: based on acquired data** **[** 14 **,** 16 **-** 19 **,** 21 **]** **and classification in Table** 2 **; population counts (P) shown on x-axis; C, D based on data for equivalent years from JMP country files** **[** 34 **]).**

Figure 1(A) and (B) summarise national percentage access to ‘preferred’ water and sanitation respectively by case study country. Colombia and South Africa show significant gaps between urban and rural areas for both water and sanitation access; however, Egypt in contrast has similar high coverage for both urban and rural areas. Figure 1(C) and (D) show the JMP estimated improved water and sanitation coverage for the same years. Despite the definitional differences between improved and ‘preferred’ classifications shown in Table 2, the JMP improved water and sanitation coverage figures are similar to ‘preferred’ service coverage for Colombia and Egypt figures and water coverage figures are similar for South Africa. In contrast, Kenya and Uganda show marked differences between our figures and JMP estimates for both water and sanitation, whilst South African figures are markedly different for sanitation only.

### Dissimilarity index

**Figure 2:**
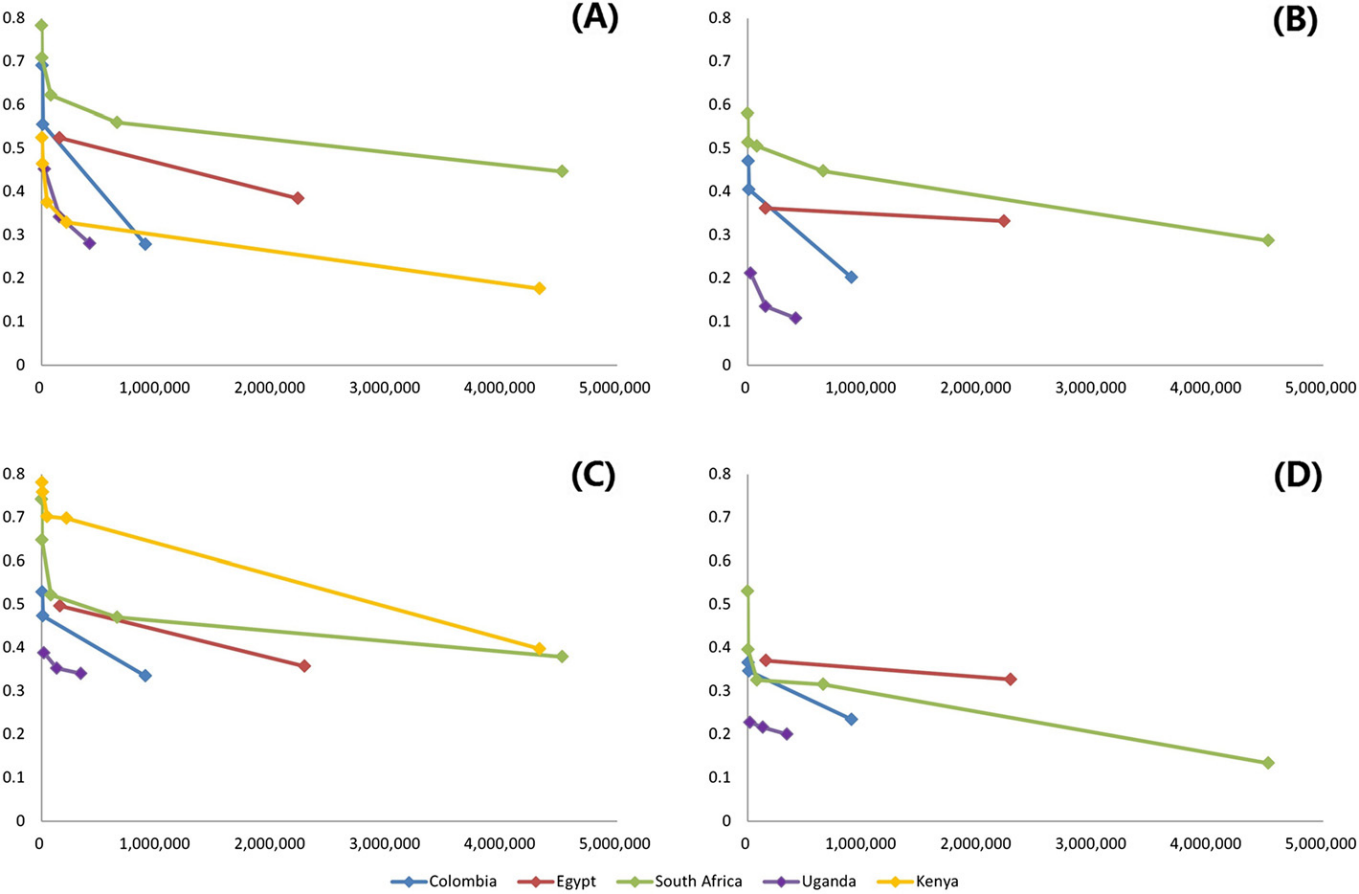
**Dissimilarity indices for water and sanitation. (A)** D for water; **(B)** D(adj) for water; **(C)** D for sanitation; **(D)** D(adj) for sanitation. The Y-axis represents the dissimilarity index value for each administrative tier and the X-axis is the median population size of the spatial units.

Figure 2 shows the values of *D* and *D(adj)* for access to drinking-water and sanitation, calculated for different administrative levels. The median population size of administrative units is used for the X-axis, since for different countries the size of administrative units at a given level (e.g. province, district, or sub-district) varies. Line graphs for the other two non-spatial variants of dissimilarity index are omitted, since the resultant values of *D*_*a*_ and *D*_a_* metrics are very close to *D,* differing by less than 0.01 in all cases. In contrast, *D(adj)* shows different patterns from the other three indices. Dissimilarity index values (excluding the non-spatial Kenya data) for all aggregation levels and variants of the index are consistently higher for piped water access in South Africa in 2001 than any of the other three countries and years (Figure 2A, B, C), with the exception of the spatially adjusted measure of inequality in sanitation access (Figure 2D). Values for all variants of the dissimilarity index decrease as areal units are aggregated together. For the same administrative level of any country, there is no direct relationship between the inequality in drinking-water access and that in sanitation access. Nevertheless, our study countries with high levels of inequality in access to drinking-water generally suffer high levels of inequality in access to sanitation as well, except for Kenya which has low levels of inequality in access to drinking-water (Figure 2A) but high levels of inequality in access to sanitation (Figure 2C). No apparent relationship is found between the dissimilarity index values and total service coverage.

### Localised dissimilarity index

Localised dissimilarity index values are illustrated using Egypt as an example, where districts (*kism*/*markaz*) form the smallest spatial unit, which are then aggregated into broader regions, known as governorates. Similar patterns were found in maps for Colombia, South Africa and Uganda (data not shown).

**Figure 3:**
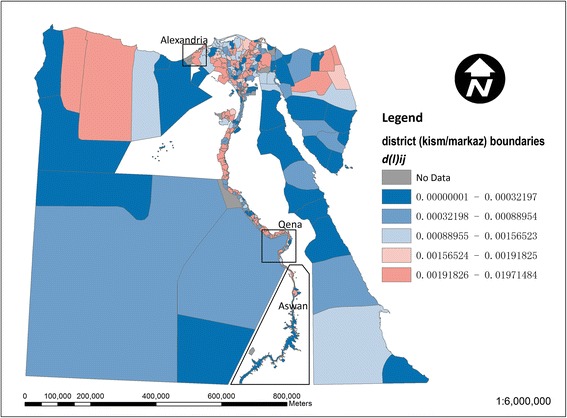
**Map of local contributions to the national dissimilarity index for drinking-water access in Egypt.** Above average contributions to inequality (values of d(l)_ij_ > d(t)_l_) are shown in red and remaining areas in blue.

**Figure 4:**
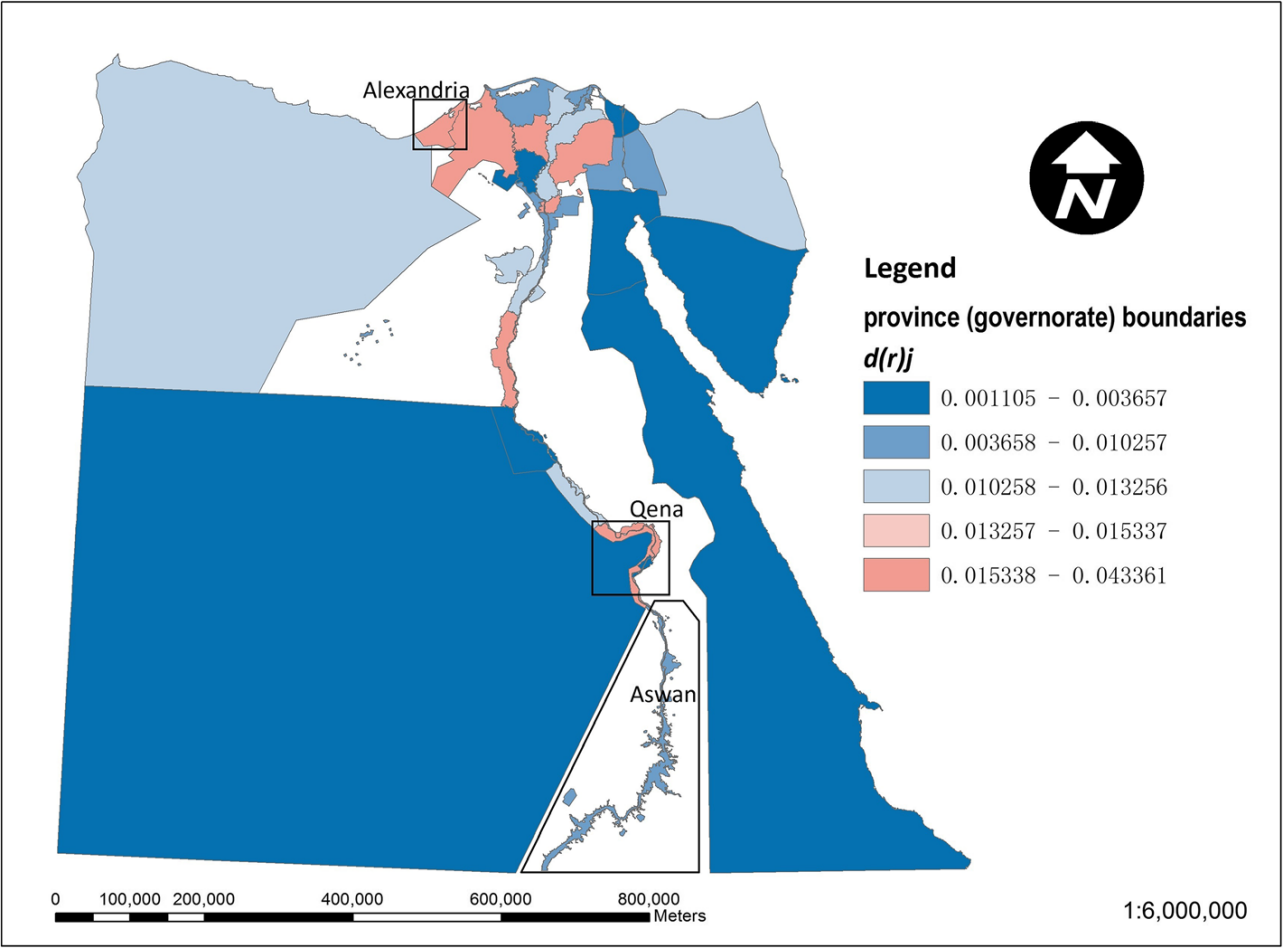
**Map of the regional component of the local dissimilarity index for drinking-water access in Egypt.** Strong contributors to inequality (values of d(r)_i_ > d(t)_r_) are shown in red and lower values in blue.

**Figure 5:**
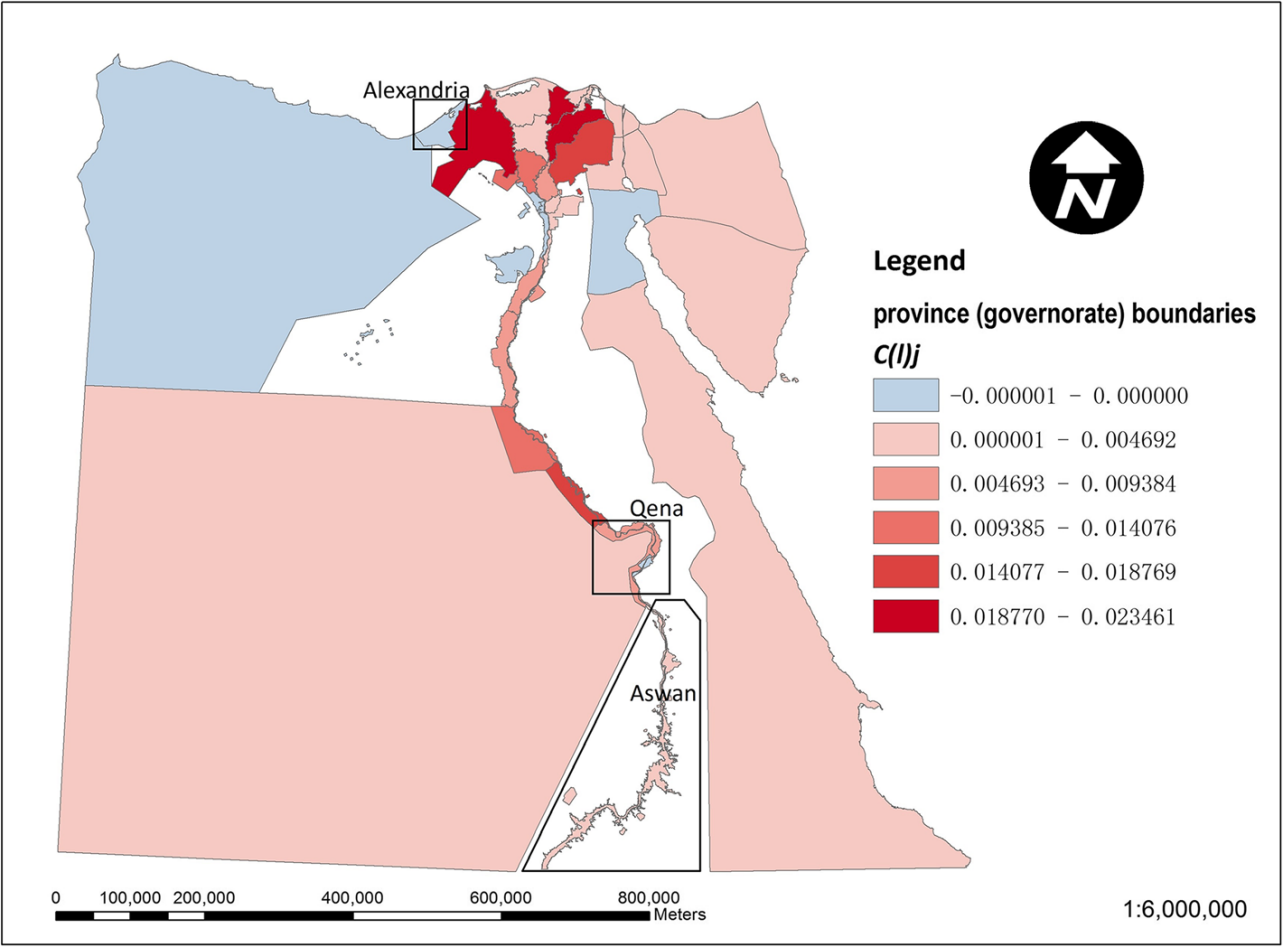
**Map of the intra-regional component of the local dissimilarity index for drinking- water in Egypt.** Strong contributors to inequality (values of C(l)_j_ > 0) are shown in red and other areas in blue).

Figure 3 shows the local contribution of each *kism*/*markaz* to the national dissimilarity index for drinking-water access (based on equation (5)). The larger *markaz* in the desert areas away from the Nile are generally below average contributors, whilst stronger contributors are noticeable particularly among urban *kism* in the northwestern Nile delta around Alexandria. This local inequality contribution in Figure 3 is further decomposed into a regional (governorate level) component in Figure 4 and an intra-regional component in Figure 5 (based on equations (6) and (9)). In Figure 4, in terms of the regional component of inequality, many of the larger governorates in Egypt’s desert areas are below average contributors to inequality, whilst stronger contributions are apparent in the Nile delta and Qena and Aswan governorates along the Nile’s southern banks. In Figure 5, strong localised, intra-regional contributions to inequality are apparent in patches of both the western and eastern Nile delta.

**Figure 6:**
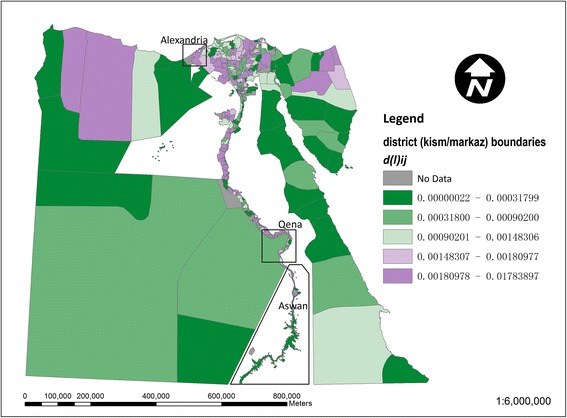
**Map of local contributions to the national dissimilarity index for sanitation access in Egypt.** Above average contributors (values of d(l)_ij_ > d(t)_l_) are shown in red and remaining areas in green.

**Figure 7:**
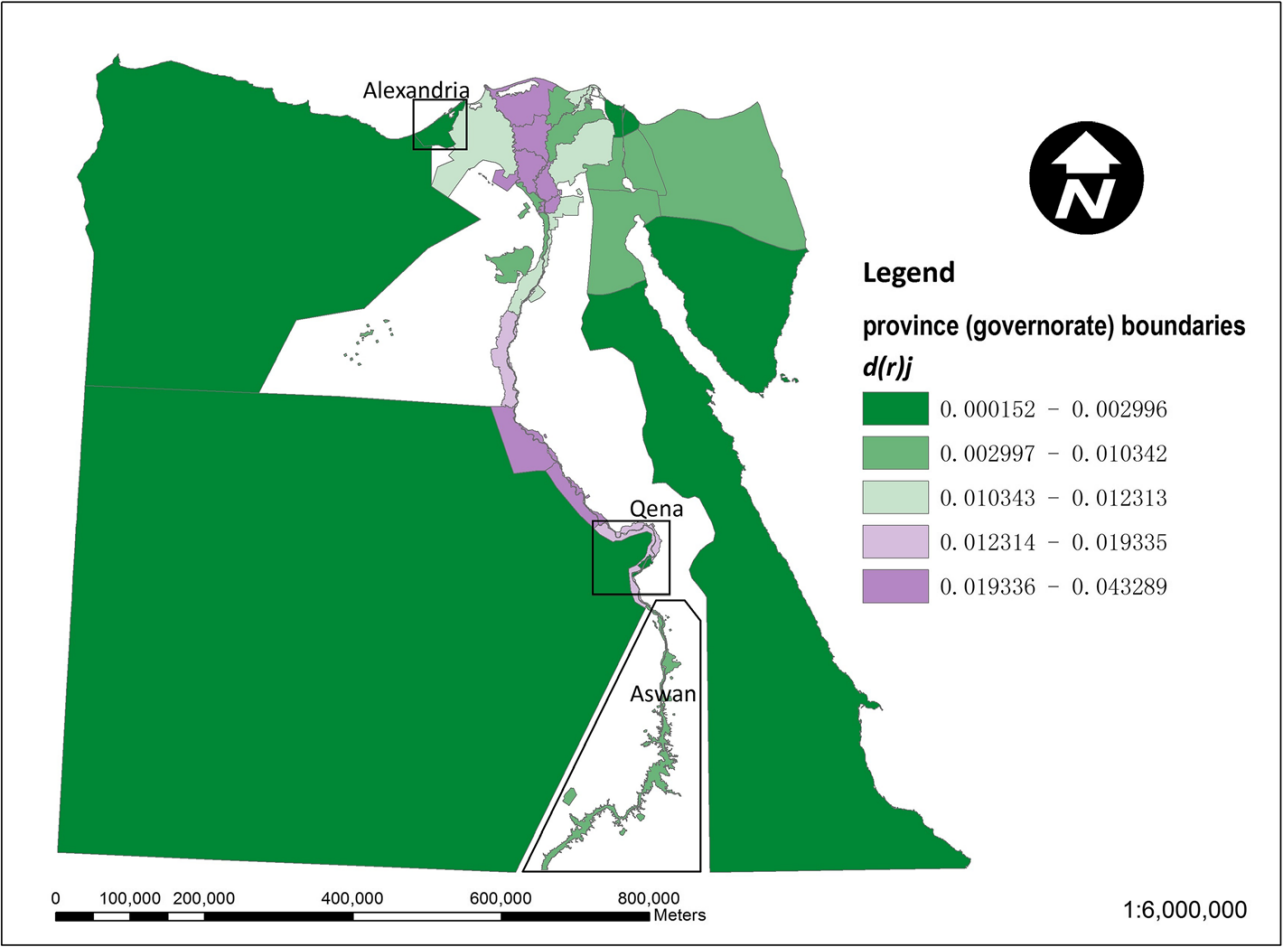
**Map of the regional component of the local dissimilarity index for sanitation access in Egypt.** Strong contributors to inequality (values of d(r)_i_ > d(t)_r_) are shown in red and lower values in green.

**Figure 8:**
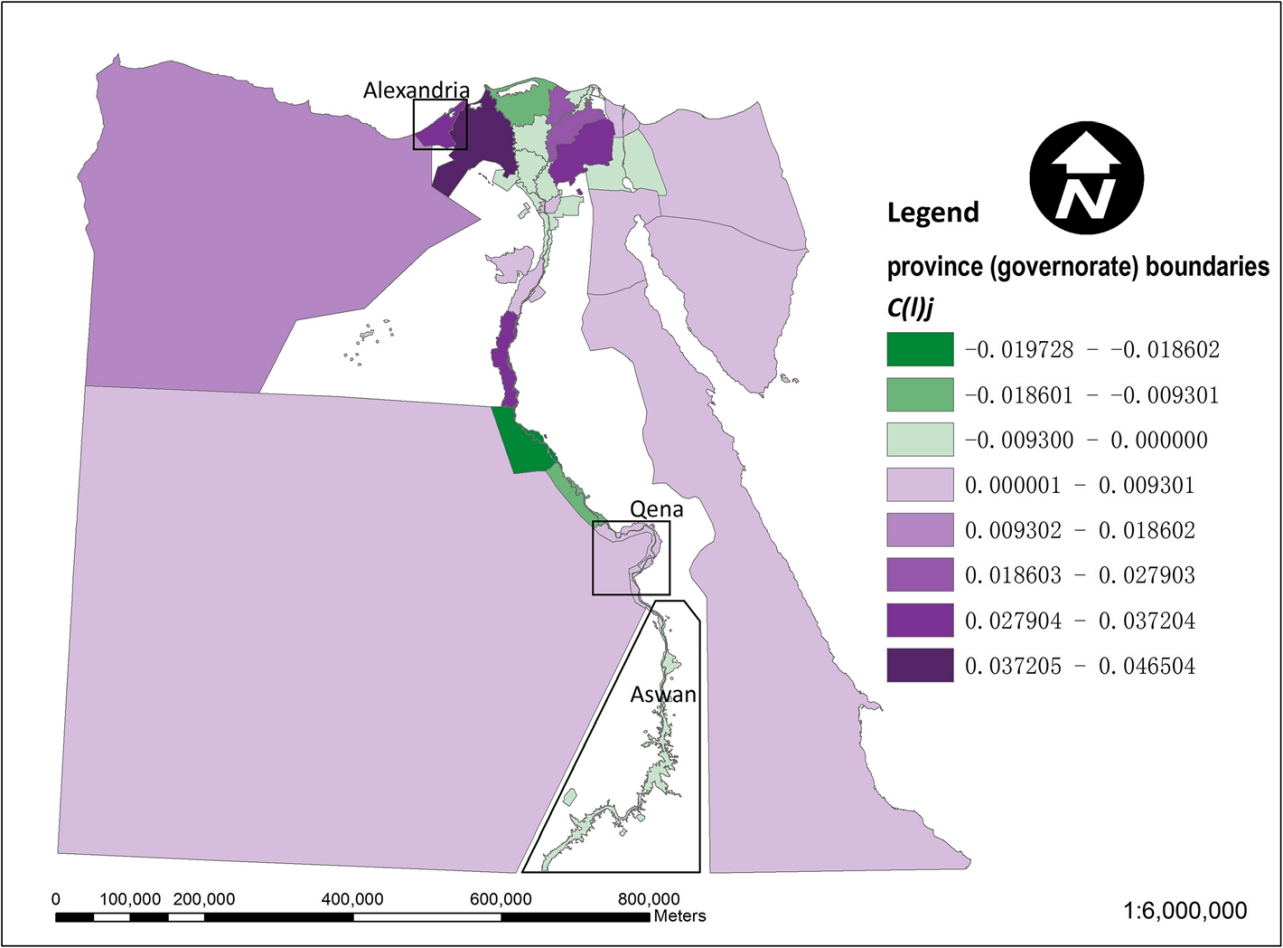
**Map of the intra-regional component of the local dissimilarity index for sanitation access in Egypt.** Strong contributors to inequality (values of C(l)_j_ > 0) are shown in red and other areas in green).

Figure 6 shows the local contribution of each *kism*/*markaz* to the dissimilarity index value for access to sanitation. The pattern in Figure 6 is broadly similar to that for drinking-water access in Figure 3, with many of the same *kism*/*markaz* contributing towards the national dissimilarity index value for sanitation. This sanitation-related contribution is again broken down into a regional (governorate level) component in Figure 7 and an intra-regional component in Figure 8. In Figure 7, in terms of the regional component of inequality, many of the larger governorates in Egypt’s desert areas are again below average contributors to sanitation-related inequality. In Figure 8, as with sanitation, there are strong localised, intra-regional contributions to inequality in some *kism* / *markaz* in both the eastern and western tributaries of the Nile delta.

**Figure 9:**
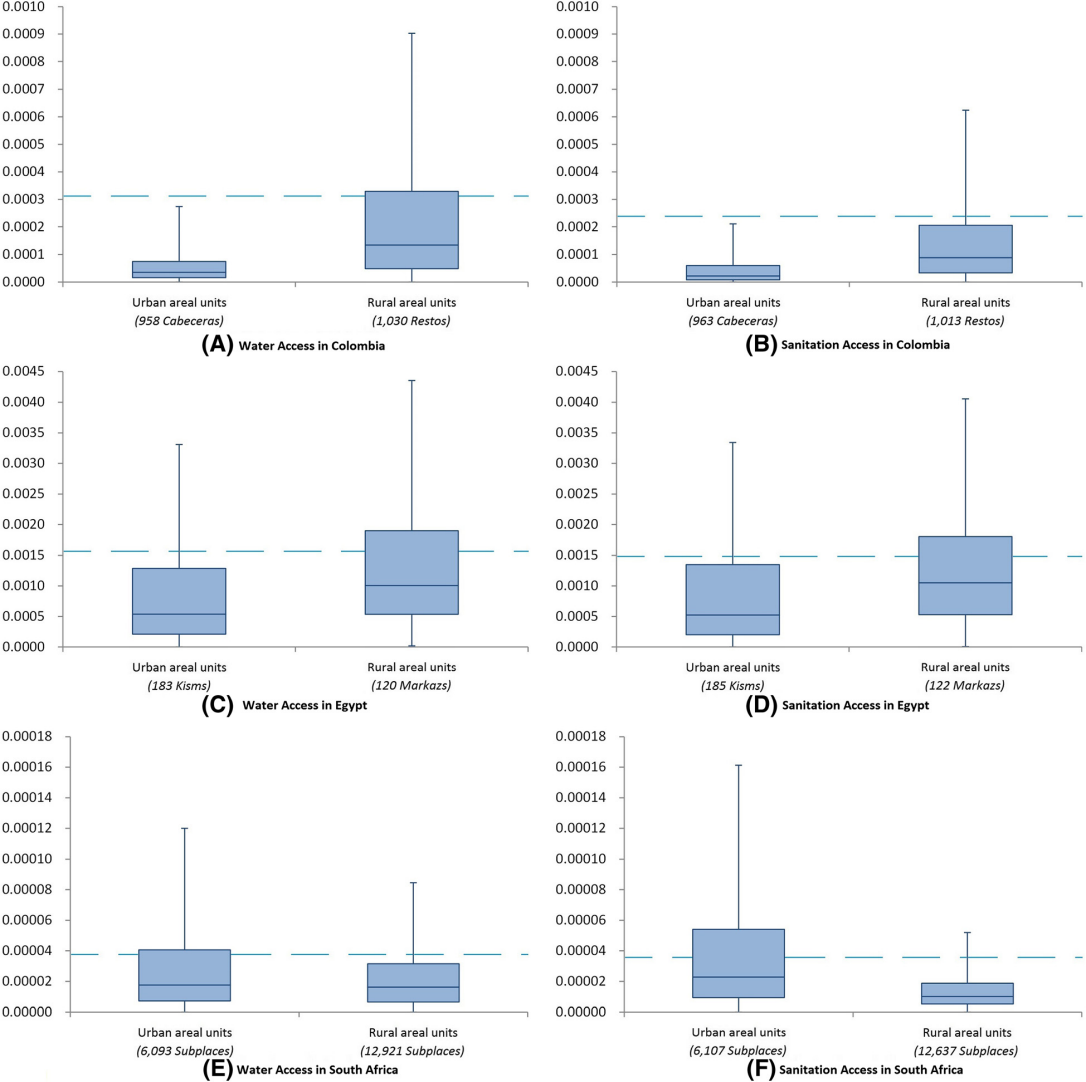
**Urban versus rural boxplots of local contributions (d**_**i**_ **), which sum to the national dissimilarity index.** Values of local d_i_ are shown on the y-axis, whilst the numbers of contributing areal units is shown on the x-axis. Separate graphs are presented for drinking-water and sanitation access in different countries: **(A)** drinking-water access in Colombia; **(B)** sanitation access in Colombia; **(C)** drinking-water access in Egypt; **(D)** sanitation access in Egypt; **(E)** drinking-water access in South Africa; **(F)** sanitation access in South Africa. Outlying values are not shown, and the bottom and top of the box are the 25^th^ and 75^th^ percentiles respectively; the superimposed dashed line represents the average local contribution, d(t).

Figure 9 shows the local contribution of each of the most disaggregated areal units to national inequality in Colombia, Egypt, and South Africa (based on equation (5)). These local contributions sum to the overall national dissimilarity index value, so their magnitude is smallest for South Africa, which has the greatest number of contributing areal units. For both water and sanitation, rural areas contribute more to national inequality than urban areas in Colombia and Egypt (Figure 9*A*, *B*, *C*, *D*), but this pattern does not hold true for South Africa (Figure 9*E*, *F*).

## Discussion

### Patterns in spatial inequality in access to water and sanitation

In all five study countries, all dissimilarity index values increased as data were disaggregated into progressively smaller spatial units. Localised pockets of population with limited or no water and sanitation services therefore lead to higher dissimilarity index values for the most disaggregated data. This pattern reflects that observed elsewhere for multiple deprivation, which typically includes a service access component. In high income countries such as the UK, highly localised pockets of material deprivation have been reported, particularly in rural areas [35]. It thus seems plausible that the more aggregated data for Egypt may mask localised variation in service access at the most disaggregated (*shyakha*) level. More generally, this suggests the population size of spatial units should be taken into account when making international comparisons based on census-derived estimates of geographic inequality in water and sanitation. More widespread publication of disaggregated census data, subject to appropriate disclosure control, and their subsequent use for measuring inequality, would overcome this issue.

Evidence from across sub-Saharan Africa suggests that the magnitude of water and sanitation-related inequalities is similar [8]. Here, a similar pattern emerged, with sanitation-related inequalities being higher than those relating to water access for Kenya, at departmental level in Colombia, and at district and county levels for Uganda, but water access-related inequality was greater for other countries and administrative levels. The large dissimilarity index values for piped water access in South Africa in 2001 relative to most of our other case study countries are unsurprising, given that income inequality there is among the highest in the world [36]. Responsibility for water services is devolved to the municipality level in South Africa and there is known to be great variation in capacity among different municipalities [37], which may further exacerbate geographic gaps in water and sanitation service access. Since income inequality is estimated to have grown in the immediate post-apartheid period [36], extending this analysis to include the 2011 South African census could shed valuable insights into trends in service access.

### Application of the dissimilarity index to water and sanitation

Wong [38] suggests that spatial dissimilarity metrics such as *D(adj)* can be even more scale-dependent than non-spatial metrics such as the classic *D*. However, in this study, the values of *D(adj)* for Egypt, Colombia and rural Uganda changed more gradually with increasing disaggregation, relative to the values of *D. D(adj)* is just one of several variants of the dissimilarity index that measure the gap in coverage between one area and its immediate neighbours, rather than relative to overall national coverage. For example, more sophisticated spatial measures of ethnic segregation take into account the length of shared boundary, and perimeter-area ratio [39]. Many of these measures seek to assess the potential for interaction between different ethnic groups and so do not translate readily into analyses of service availability. Thus, whilst *D(adj)* may be helpful in identifying more localised discrepancies in service coverage (e.g. the close juxtaposition of areas with high and low service coverage), these other inequality measures may be less applicable to analysis of water and sanitation coverage.

In our implementation of the dissimilarity index, we considered only two population groups, namely those with and without access to the water and sanitation classes shown in Table 2. Since the dissimilarity index has been modified to enable analysis of more than two groups [40], it would be possible to examine spatial inequality in finer-grained water and sanitation classifications. Such classifications include the water ‘ladder’ [24], which distinguishes access to piped water on premises from other forms of ‘improved’ water provision, as well as surface water from other forms of ‘unimproved’ water provision. We applied the dissimilarity index to population groups in an ecological study and thus our findings are subject to the generic weaknesses of such studies. For example, we were unable to explore the inter-relationship between household socio-economic status and service access in drawing on aggregated rather than micro-data.

### Census data and inequality in water and sanitation access

Since they are based on a complete (or nearly complete) enumeration of population, census data have the advantage of allowing greater geographic disaggregation of water and sanitation data, relative to household surveys. In contrast, disaggregation of household survey data requires estimation of local coverage via techniques such as Bayesian conditional autoregressive modelling [8] and local variation in service coverage may be smoothed by such techniques. However, our five-country study suggests that patterns of inequality for broad regional units do often reflect inequality in service access at a more local scale. This implies household surveys designed to estimate province-level service coverage can provide insights into geographic inequality.

Despite its ability to provide geographically disaggregated data, there are difficulties in measuring water and sanitation coverage via population census. Although it would be possible to do so by analysing temporal trends from a wider range of household surveys and censuses [7,8], we have not adjusted estimates to account for the differing dates of census enumeration between the five study countries. Aside from the data currency issues resulting from decadely censuses, census enumeration dates are more likely to be in the dry than wet season in low and middle income countries [41], with consequent seasonal bias in rainwater coverage estimates, for example. Despite efforts to harmonise definitions [42], internationally census questions and responses concerning household use of water and sanitation are less standardised and typically less detailed than those in household surveys such as the DHS, where a standardised core set of questions on water, sanitation and hygiene is used [43]. Water and sanitation categories are often also grouped together in geographically disaggregated census data. This is reflected in the inconsistency in the definitions of water and sanitation access used for each country in the analysis presented here. For example, in South Africa we examined inequalities in piped water access, whereas in Kenya and Colombia we examined inequalities in ‘improved’ water access (which includes boreholes, rainwater and protected wells in addition to piped water). Similarly, in Kenya and Uganda it was not possible to differentiate between pit latrines with and without a slab, which is used to distinguish ‘improved’ from ‘unimproved’ sanitation in the JMP classification.

Future work could potentially use household survey data in combination with census data to estimate water and sanitation access at a more disaggregated level, drawing on the spatial detail of censuses and internationally standardised format and wider set of variables in survey data. For example, small area estimates of poverty have been generated by enriching census data using relationships estimated from household surveys that predict income, which is not covered by censuses [44]. Analogously, these same techniques could potentially be applied to a more comprehensive metric of water or sanitation access that incorporates other aspects such as distance to facility, supply interruptions.

In addition, studies of health inequalities have pointed towards two data-related effects that can confound the analysis of real, underlying variation in population access to services, namely a scale effect and an aggregation effect [11]. Scale effects occur as intra-areal variation in access to services is averaged out, as aggregate summary statistics are calculated for progressively larger areas. Aggregation effects relate to the nature of the boundaries used to aggregate data, and such effects typically depend on how homogenous different administrative areas are. Some boundary systems may bring together deprived and more affluent neighbourhoods within a single administrative district, for example where pockets of informal settlement have developed alongside older, more established suburbs. Such boundary systems are more likely to mask localised pockets where people lack access to water and sanitation, compared with those that are more socio-economically homogenous. In some high income countries, census boundaries are designed by algorithm to be homogenous [45]. These algorithms are beginning to be applied to middle income countries, with a set of ‘datazones’ being designed for South Africa based on an explicit social homogeneity criterion [46]. However, such boundaries are unlikely to come into widespread use in low and middle income countries in the short to medium term. Therefore, any aggregation effect depends on the specific local context and how national boundaries were formulated.

Short format census questions may also not capture the use of multiple water sources for different domestic purposes [47]. Similarly, the difficulties with the ‘improved’/‘unimproved’ classification of water and sanitation types are also well recognised, given the classification’s failure to account for the quality and quantity of water supplied [48,49] as well as affordability and sustainability issues. The limited evidence from household surveys suggests that at least in some settings, measured inequality in access to safe drinking-water would be even greater were water quality to be taken into account [9].

Aside from drawing on population censuses, there may be potential to use the results of water point mapping [50] for assessing inequality, as attempted here for Uganda. However, such inventories require assumptions to be made about the population served by each water point and may omit less commonly encountered types of water source, such as rainwater. Furthermore, water point mapping typically measures service availability, rather than service use.

## Conclusion

For our five case study countries, measured inequality in water and sanitation access was consistently greater when calculated for small spatial units. However, the relative rankings of our study countries in terms of inequality in water and sanitation access generally remained unchanged when calculated based on geographic units of different sizes, although Kenya’s ranking changed when measuring inequality in improved water. Consistent with the high income inequality there, South Africa in 2001 had the greatest measured level of inequality in piped water access and high inequality in sanitation access. Kenya in 2009 had the greatest level of inequality in access to any form of sanitation facility but very low inequality in ‘preferred’ water access.

Despite the geographic detail that census data provide, substantial analytical effort would be required to enable the dissimilarity index to be suitable for census-based, international comparisons of geographic inequality in water and sanitation access. As noted earlier, water and sanitation categories would need to be harmonised across countries and census years. Trends in dissimilarity index values from successive censuses would need to be calculated, so as to facilitate adjustment to account for international variation in census enumeration dates. Finally, further empirical adjustment would be required to account for international variation in the population sizes and nature of census geographic units.

This analysis suggests that census data can be particularly useful in identifying variation in water and sanitation use at different geographic scales since they are based on a complete (or near complete) enumeration of population. However, care is required in interpreting the resultant inequality metrics, both as census questions and responses may differ from country to country and because of international variation in the size and homogeneity of census areal units. Our results suggest that the classic dissimilarity index, *D*, may be a straightforward and appropriate national metric of inequality in service access, and correction for scale-related random effects may be unnecessary. There is scope to extend such analysis further to examine trends over time and to examine the spatial distribution of multiple tiers of service access, rather than simply ‘haves’ and ‘have nots’.
